# Phosphorylated proteomics-based analysis of the effects of semaglutide on hippocampi of high-fat diet-induced-obese mice

**DOI:** 10.1186/s13098-023-01023-y

**Published:** 2023-03-30

**Authors:** Xiaoyi Chen, Liang Ma, Kexin Gan, Xiaoyu Pan, Shuchun Chen

**Affiliations:** 1 Graduate School of Hebei North University, Zhangjiakou, China; 2 Department of Endocrinology, Hebei General Hospital, Shijiazhuang, China; 3 Department of Neurology, Hebei General Hospital, Shijiazhuang, China

**Keywords:** Semaglutide, Axonal growth, Learning memory, Phosphorylated proteomics, Obesity

## Abstract

The aim of this paper was to investigate the effects of semaglutide on phosphorylated protein expression, and its neuroprotective mechanism in hippocampi of high-fat-diet-induced obese mice. In total, 16 obese mice were randomly divided into model group (H group) and semaglutide group (S group), with 8 mice in each group. In addition, a control group (C group) was set up comprising 8 C57BL/6J male normal mice. The Morris water maze assay was conducted to detect cognitive function changes in the mice, and to observe and compare body weight and expression levels of serological indicators between groups after the intervention. Phosphorylated proteomic analysis was performed to detect the hippocampal protein profile in mice. Proteins up-regulated twofold or down-regulated 0.5-fold in each group and with t-test *p* < 0.05 were defined as differentially phosphorylated proteins and were analyzed bioinformatically. The results showed that the high-fat diet-induced obese mice had reduced body weight, improved oxidative stress indexes, significantly increased the percentage of water maze trips and the number of platform crossings, and significantly shortened the water maze platform latency after semaglutide intervention. The phosphorylated proteomics results identified that 44 overlapping proteins among the three experimental groups. Most of the phosphorylated proteins identified were closely associated with pathways of neurodegeneration-multiple diseases. In addition, we identified Huntington, Neurofilament light chain, Neurofilament heavy chain as drug targets. This study demonstrates for the first time that semaglutide exerts neuroprotective effects by reducing HTT Ser1843, NEFH Ser 661 phosphorylation and increasing NEFL Ser 473 phosphorylation in hippocampal tissue of obese mice.

## Introduction

Obesity is a common metabolic disease and a major risk to human health. According to the latest projections, by 2030, there will be 2.16 billion overweight and 1.12 billion obese people worldwide [32]. In addition to increasing the risk of diabetes, cardiovascular disease, stroke and certain cancers, obesity is also closely associated with central nervous system hypofunction. Studies have shown that high-fat-diet-induced obese mice exhibit cognitive dysfunction, depression-like symptoms and anxiety behaviors [31]. Large population studies have also shown a linear relationship between obesity and cognitive impairment, and that obesity can increase brain damage and accelerate brain aging, thereby exacerbating neurodegenerative diseases [24]. The postulated pathways mediating these effects include insulin resistance, the gut-brain axis, systemic mediators, and central inflammatory processes. However, the exact mechanisms underlying the link between obesity and risk of cognitive impairment remain elusive.

Post-translational modifications such as protein phosphorylation and ubiquitination function as gatekeepers in various cellular processes [15]. It is now widely accepted that phosphorylation of abnormal proteins participates in the pathogenesis of several diseases [5, 14, 20]. Protein phosphorylation is also considered the main post-translational modification that rapidly, reversibly, and highly specifically induces or eliminates enzyme activity and promotes or disrupts protein interactions by changing protein conformation [13]. Studies have shown that activation of GSK3b (via autophosphorylation) and phosphorylation of Tau significantly contribute to neurodegeneration [5, 27]. However, the exact role of aberrant protein phosphorylation in obesity-related cognitive disorders is not fully understood.

Human glucagon-like peptide-1 (GLP-1) is an endogenous intestinal glucagon peptide hormone and an important treatment target in type 2 diabetes. GLP-1 and its analogues can cross the blood–brain barrier to affect physiological activities and neurogenesis in brain tissues. Its target GLP-1 receptors are expressed throughout the cortex and hypothalamus of the central nervous system [12]. These provide favorable conditions for the treatment of CNS lesions with GLP-1 analogs. Semaglutide is a novel long-acting GLP-1 receptor agonist with about 94% structural homology to natural human GLP-1. It selectively binds to GLP-1 receptors to activate the corresponding receptors [30]. Animal models studies have shown that Alzheimer's disease [6], Parkinson's disease [37] and epilepsy [33], stigmasteride can protect neuronal cells by inhibiting glial cell activation, neuroinflammation, and oxidative stress. However, the neuroprotective effect of semaglutide in obesity-related cognitive disorders and its mechanism of action have not been investigated.

In this study, we used high-fat diet-induced obese mice to investigate the effects of semaglutide on the expression of hippocampal-associated phosphorylated proteins and its neuroprotective effects in mice with phosphorylated proteomics to identify important pathways and therapeutic targets in obesity-related cognitive disorders.

## Materials and methods

### Animals

The experimental subjects of this study were 24 SPF-grade C57BL/6J male mice (SYXK[Jun] 2015-0004, Hebei In vivo Biotechnology Co., Ltd), 6 weeks old and weighing 17.8–21.6 g. The mice were housed in the Animal Experiment Center of Hebei General Hospital, with 4 mice per cage, and maintained at 20–25 °C, 50–55% humidity, with artificial light source simulating a circadian cycle (12 h/12 h). Eight healthy mice were randomly assigned to the normal control group (group C) and fed with regular chow (D1035, 10% energy from lipids, Beijing Huafukang Biotechnology Co., LTD, China), free food and water. The rest were fed with high-fat diet (H10060, 60% energy from lipids, Beijing Huafukang Biotechnology Co., LTD, China) for 12 weeks. An average weight increase of 20% compared with the normal-diet group was used as the criterion for obese mice. The obese mice were randomly divided into the model group (high-fat diet, group H) and semaglutide group (H+ semaglutide, group S), comprising 8 mice in each group. Mice in group S were treated with semaglutide 30 nmol/kg/d for 12 weeks whereas mice in groups H and C were treated with equal amounts of saline. The study followed the guidelines of the Guide for the Care and Use of Laboratory Animals and was approved by our Animal Ethics Committee (No. 202173). All methods below are from previous studies [7].

### Animal cognitive level testing

Morris water maze experiments (8 mice per group) were started at 24 h after the last dose. The experiment consisted of a 4-day positioning navigation test and a 1-day spatial exploration experiment in a circular pool, 1.2 m in diameter and 0.45 m in height. Positioning navigation test: The platform was placed and maintained at 1.0–2.0 cm under water. The mice in each group were put into the water facing the wall of the pool. The distance they swam from the entry point to climbing onto the platform was recorded, i.e., the escape latency distance. Spatial exploration test: The underwater platform was removed and the mice were given 60 s to explore the platform. The mice's swimming trajectory, the number of times they crossed the platform, and the percentage of time they stayed in the target quadrant were recorded and analyzed.

### Collection of blood and hippocampal tissue samples from mice

After the behavioral assay, all mice were bled from the eyeball, blood was coagulated at 4 °C for 30 min, then centrifuged at 3000×*g* for 10 min, serum was collected and set aside. after blood collection, mouse hippocampal tissue was quickly stripped at low temperature, and serum and hippocampal tissue were stored at − 80 °C.

### Lipid and pro-inflammatory factor level testing

Total cholesterol (TC), triglyceride (TG), plasma high-density lipoprotein (HDL-C) and plasma low-density lipoprotein (LDL-C) concentrations in serum samples were measured using a fully automated enzyme marker (USA). Interleukin 6 (IL-6), interleukin 1β concentrations in serum samples were determined using ELISA kits following manufacturer’s instructions (Multi Sciences Biotech Co., Ltd., Hangzhou, China).

### Measurement of superoxide dismutase activity (SOD) and lipid peroxidation

Serum SOD concentration was measured using Superoxide Dismutase Assay Kit (Nanjing Jiancheng Bioengineering Institute, China). Serum malondialdehyde (MDA) concentration, which reflects the degree of lipid peroxidation in the body, was measured using Malondialdehyde (MDA) Assay Kit (Nanjing Jiancheng Institute of Biological Engineering).

### Proteomic sample preparation

Tissue samples were ground in liquid nitrogen, and the sample lysates were mixed with protease and phosphatase inhibitors. The samples were then sonicated on ice (SCIENTZ-II D) at 80 W for 3 min. Subsequently, the samples were centrifuged twice at 12,000*g* for 10 min. The supernatant was gently aspirated, taking care not to aspirate the lower precipitate, and quantified with the BCA method. The samples were reduced and oxidized by adding dithiothreitol (5 mM, 55 °C, water bath for 30 min) and iodoacetamide (10 mM, light avoidance, 15 min), respectively, to the protein according to volume. Six times the volume of protein precipitant was added, mixed well and precipitated overnight at − 20 °C. The following day, the samples were removed and centrifuged at 4 °C and centrifugal force of 8000*g* for 10 min to collect the precipitate. Then, 50 mM ammonium bicarbonate was added to each sample to re-solubilize the precipitate. Trypsin (trypsin: protein = 1:50) was then added and samples were incubated at 37 °C left overnight. The enzymatically digested samples were desalted, lyophilized, and stored at − 80 °C.

### Phosphorylated peptide enrichment

For the enrichment of phosphorylated peptides, IMAC enrichment was used, which consisted of five steps: suspension of peptide samples, equilibration, binding of phosphorylated peptides, washing column, and elution column. Firstly, lyophilized peptide samples were completely suspended in 200 µL of Binding/Wash Buffer. Then, column was equilibrated as follows: (1) the bottom cap of the rotating column was removed and the screw cap loosened; (2) the column was placed in a 2 mL microcentrifuge tube for centrifugation at centrifugal force of 1000*g* for 30 s; (3) Then, 200 µL of Binding/Wash Buffer was added and centrifuged for 30 s at the same centrifugal force with the flow-through discarded (this step was repeated once) and (4) a white luer plug was used to plug the bottom of the column, which was then placed in an empty microcentrifuge tube. To reconjugate the phosphorylated peptide, the following steps were performed: (1) 200 µL of the suspended peptide sample was added to the equilibrated centrifuge column and the screw cap closed; (2) while holding the screw cap, the bottom plug was gently tapped for 10 s to suspend the sample and mix it well with the resin; (3) the mixture was then incubated for 30 min and then the resin was gently mixed every 10 min as described in step 2; (4) the bottom plug and screw cap were carefully removed; and (5) the column was placed in a microcentrifuge tube and centrifuged for 30 s at a centrifugal force of 1000*g*. The flow-through solution was discarded. To wash the column, the following steps were performed: (1) the column was washed with 200 µL of Binding/Wash buffer and centrifuged for 30 s at 1000*g* (this step was repeated twice); (2) the column was washed again with 200 µL of LC–MS grade water and centrifuged once at the same rate. For the final elution of the column, (1) the column was transferred to a new microcentrifuge tube: (2) 100 µL of elution buffer was added to the column and centrifuged for 30 s at a centrifugal force of 1000*g* (this step was repeated once) and (3) samples were concentrated by vacuum drying and centrifugation.

### Liquid chromatography-mass spectrometry (LC–MS) analysis

The mobile phase A was dissolved by liquid chromatography and separated using an EASY-nLC1200 ultra-high performance liquid phase system (Thermo Fisher Scientific Inc., USA). The flow rate was set at 300 nL/min and linear gradient was 60 min (0–66 min, 3–27% B; 66–73 min, 27–46% B; 73–84 min, 46–100% B; 84–90 min, 100% B; mobile phase A = 0.1% FA in water and B = 80% ACN/0.1% FA in water). The chromatographically separated samples were then analyzed using the timsTOF Pro mass spectrometer (Thermo Fisher Scientific Inc., USA). Data were acquired under the following mass spectrometry conditions: capillary voltage of 1.5 kV, drying gas temperature of 180 °C, drying gas flow rate of 3.0L/min, mass spectrometry scan range of 100–1700 m/z, ion drip range of 0.75–1.4Vs/cm2 and collision energy range of 20–59 eV.

### Database search

A database search was performed using Maxquant software (http://www.maxquant.org/) to obtain detailed biological information on the proteins detected with mass spectrometry. The mass error tolerance of the primary parent ion was set at 20 ppm and 10 ppm for First search and Main search, respectively. In addition, the mass error tolerance of the secondary fragment ion, cysteine aminomethylation and dynamic modification were set at 0.5 Da; fixed modification and oxidation of methionine, acetylation of N-terminal protein and phosphorylation of Ser, Thr, and Tyr, respectively. The false positive rate was set at 0.5 Da. For phosphorylation, the false positive rate of peptide segment identification was controlled below 1%.

### Bioinformatics analysis

The resulting differentially phosphorylated proteins were annotated based on Uniprot's annotation information using Gene Ontology (GO) for GO annotation and the Kyoto encyclopedia of genes and genomes (KEGG) for metabolic pathway enrichment analysis. Proteins were considered differentially phosphorylated when the protein abundance differed by a factor of 2 or more and the difference was significant at *p* < 0.05.

### Network pharmacology construction

To visualize protein-pathway networks and reveal key proteins, a network was constructed using Cytoscape 3.9.1 (Cytoscape Consortium, CA, USA).

### Statistical analysis

SPSS.25 statistical software was used to analyze the experimental data. All results were expressed as "mean ± standard error". For analysis of locomotor navigation experiment data, repeated measures ANOVA was used. Metabolic data were analyzed using one-way ANOVA, with post hoc tests using the LSD test. In addition, non-parametric tests were used. A *p*-value < 0.05 was defined as a statistically significant difference. All graphs were plotted using Graphpad 8.0.

## Results

### Evaluation of metabolic status of experimental mice

**Fig. 1:**
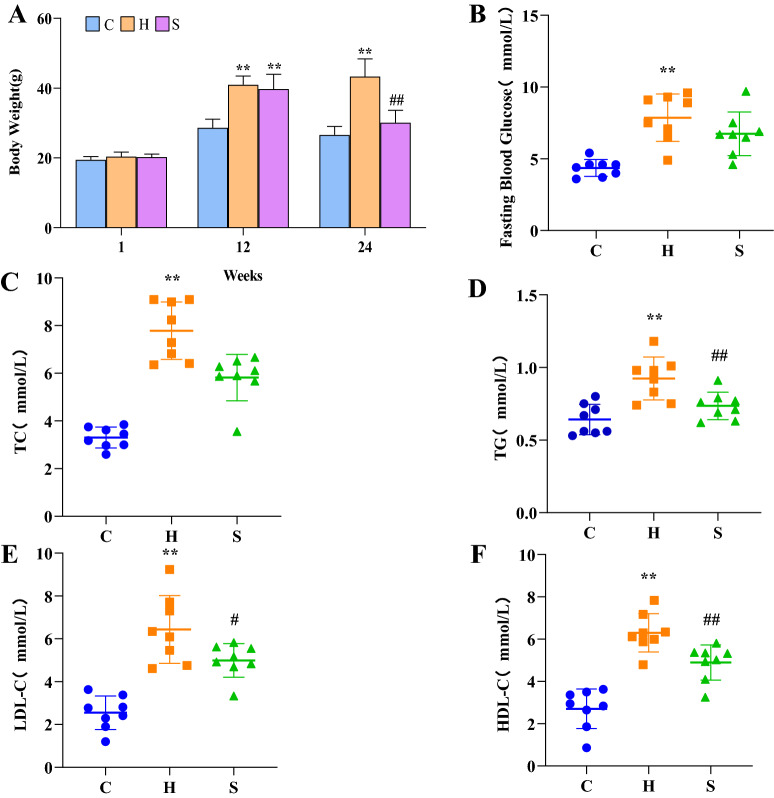
Comparison of various metabolic indices in the three groups of mice. **A** Long-term and regular high-fat diet led to weight gain in group H and S mice. The weight of mice in group S decreased significantly after semaglutide treatment. Comparison of FPG (**B**), TC (**C**), TG (**D**), LDL-C (**E**) and HDL-C (**F**) levels in mice among the three groups at the end of 24 weeks; ^*^p < 0.05 and ^**^p < 0.01 vs. C; ^#^*p* < 0.05 and ^##^*p* < 0.01 vs. H

At week 0, there was no statistically significant difference in body weight of mice in each group (*p* > 0.05). After 12 weeks of modeling, the body weight of mice in the model group was significantly higher than that in the normal group (*p* < 0.01), indicating that the mice in the model group successfully acquired an obese phenotype (Fig. 1). After treatment, compared with those in the model group, body weight, FPG, TC, TG, LDL-C and HDL-C levels of mice in the semaglutide group were all lower but to different degrees (Fig. 1).

### Effect of semaglutide on serum IL-1, IL-6, SOD and MDA levels

**Fig. 2:**
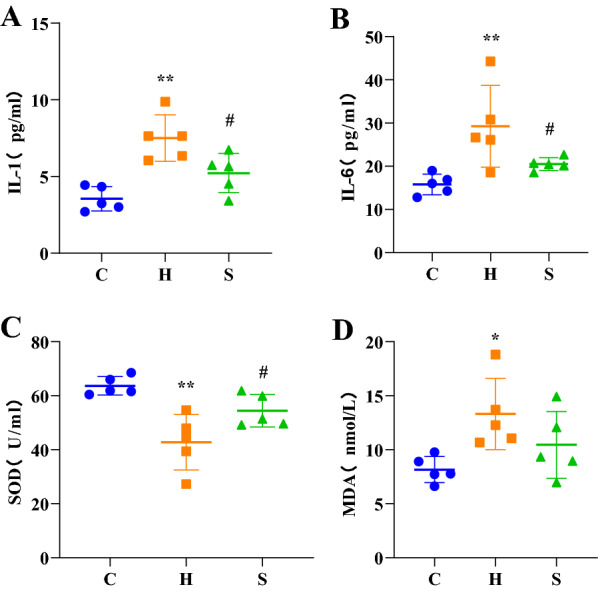
Comparison of serum IL-1 (**A**), IL-6 (**B**), SOD (**C**) and MDA (**D**) concentrations in three groups of mice. ^*^p < 0.05 and ^**^p < 0.01 vs. C; ^#^*p* < 0.05 and ^##^*p* < 0.01 vs. H

As shown in Fig. 2, in group H, the levels of IL-1, IL-6 and MDA were significantly higher than those in the control group (all *p* < 0.01), whereas the levels of SOD were significantly lower than those in the control group (*p* < 0.01). Compared with group H, group S mice showed significantly lower serum levels of IL-1, IL-6 in but significantly higher levels of SOD (all *p* < 0.05). And MDA levels decreased in group S compared with group H, but there was no statistical difference (P > 0.05).

### Effects of semaglutide on cognitive function of obesity model mice

**Fig. 3:**
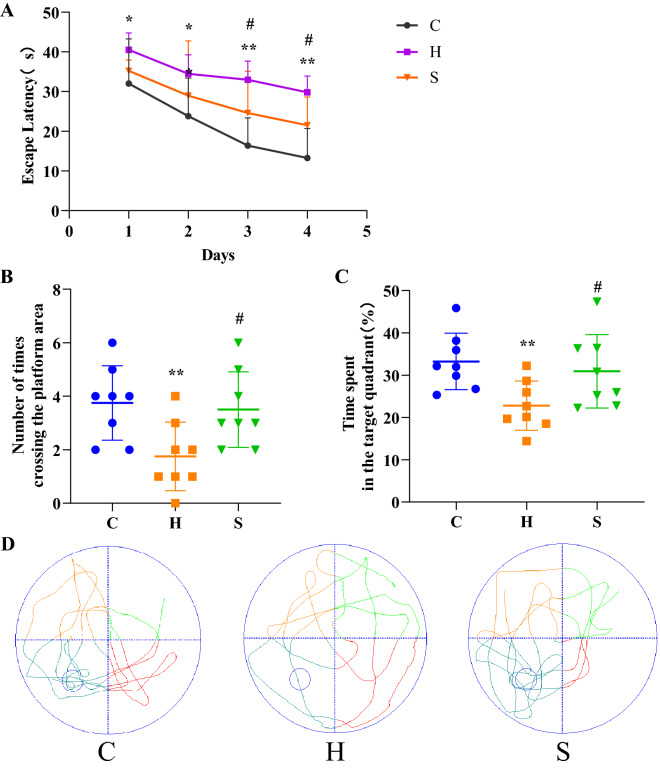
Semaglutide also improved learning and memory abilities of obese mice, n = 8 for each group. **A** Platform latency in three groups of mice in the locomotor navigation test. **B** Change in time spent in the target quadrant (%) on day 5. **C** Number of times the corresponding platform position was passed on day 5. **D** Swimming paths of the three groups of mice on day 5.^*^p < 0.05 and ^**^p < 0.01 vs. C; ^#^*p* < 0.05 and ^##^*p* < 0.01 vs. H

In the positioning navigation test performed from day 1 to day 4, the platform latency of all groups of mice gradually decreased with increasing training days. The platform latency of mice in the model group was significantly longer compared with the normal group (days 1 and 2: *p* < 0.05; days 3 and 4: *p* < 0.01) (Fig. 3A). In the spatial exploration test, the number of platform crossings and platform dwell time of mice in the model group were significantly less than those in the normal group (all *p* < 0.01), with disorganized swimming trajectory, (Fig. 3B–D). Interestingly, semaglutide significantly improved the cognitive function of obese mice. Semaglutide restored platform latency, number of platform crossings and percentage of platform dwell time (*p* < 0.05) (Fig. 3B, C).

### Phosphoproteomic analysis by 4D-Labelfree quantitative technology

**Fig. 4:**
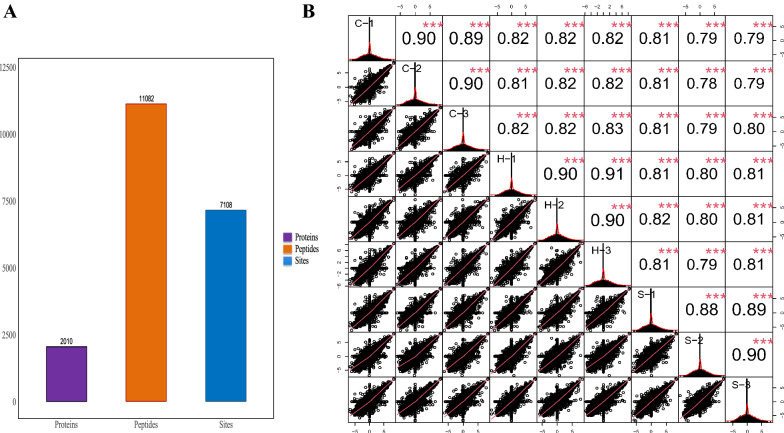
**A** Statistical information on the phosphorylated proteins/peptides/sites. **B** Upper triangle (upper right of the diagonal line); numbers indicate the correlation values of the two samples,^*^ indicates the significance level (^*^p < 0.05, ^**^p < 0.01, ^***^p < 0.001); the lower triangle (lower left of the diagonal line) gives the scatter plot of the expression values of the two samples, the red curve is the fitted trend, the higher the slope the stronger the correlation between the two samples; the diagonal line is the sample self-expression distribution graph

To investigate whether semaglutide affects protein phosphorylation in the hippocampus of high-fat-diet-induced obese mice, we analyzed the phosphorylated proteome of C57BL/6J male mice fed with normal diet, high-fat diet, and high-fat diet + semaglutide administration conditions using IMAC enrichment technique combined with high-throughput mass spectrometry. We identified a total of 7108 phosphorylation sites, which were distributed on 11,082 phosphorylated peptide fragments belonging to 2010 proteins, as shown in Fig. 4A.

Among the 7108 phosphorylation sites identified, those with localization probability ≥ 0.75 and delta score ≥ 8 card values were selected for analysis to obtain more reliable phosphorylation sites. As shown in Fig. 4B, sample correlation analysis was performed for candidate sites to reflect the grouping situation and reproducibility issues between samples by measuring the correlation degree between samples. The results showed strong the correlations within group S (S1 vs. S2 vs. S3), group H (H1 vs. H2 vs. H3) and group C (C1 vs. C2 vs. C2), which proved the reproducibility of the experiment.

### Differential screening of phosphorylation sites

**Fig. 5:**
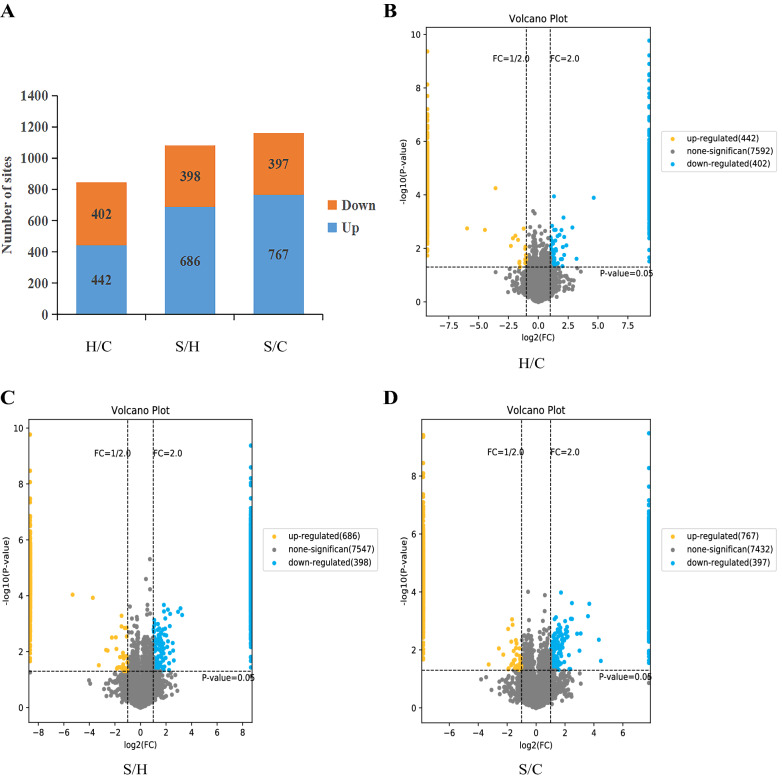
Overall distribution of differentially expressed loci. **A** Histogram of differentially expressed loci in the three groups; **B**–**D** volcano plot of differentially expressed proteins in the three groups. The horizontal axis represents log2 (FC), the vertical axis represents −log10 (*p*-value) and values closer to the center indicate smaller differences. Up-regulated and down-regulated differentially expressed proteins are indicated by blue dots and yellow dots, respectively

In the bioinformatics analysis of the phosphorylated proteome, 3092 differentially phosphorylated protein loci were identified as up-regulated by ≥ twofold foldchange or down-regulated by ≤ 0.5-fold foldchange and *p* < 0.05 (Fig. 5A). Among these differentially phosphorylated protein sites, 442, 686 and 767 sites were up-regulated whereas 402, 398 and 397 sites were down-regulated in the H/C group, S/H group, and S/C group, respectively. The volcanoes were plotted for differentially phosphorylated proteins (Fig. 5B–D).

### Annotation of the function of phosphorylated protein profiles

**Fig. 6:**
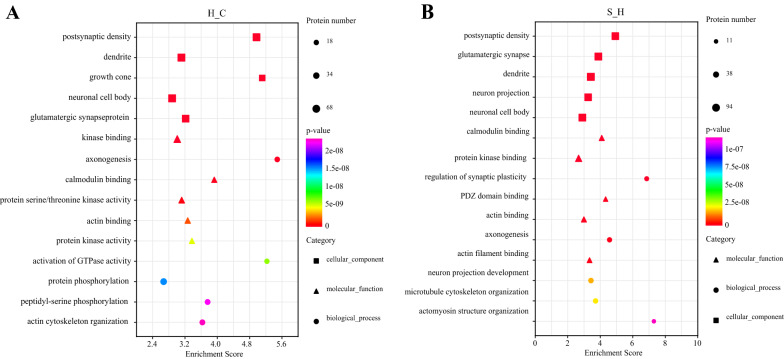
GO annotation of the proteins corresponding to the two groups of differential sites **A** H/C and **B** S/H according to cellular composition, molecular function, and biological processes. Red color indicates stronger enrichment (the darker the red color, the stronger the enrichment) and purple color indicates weaker enrichment (the lighter the purple color, the weaker the enrichment)

Based on the data from hippocampal phosphorylation proteomics, we performed functional annotation of the identified phosphorylated proteins. First, GO entries corresponding to protein numbers greater than 1 in the three classifications of cellular components, molecular functions, and biological processes were screened. Bubble maps were made for each of the five entries and ordered from the largest to smallest by the -log10P-value corresponding to each entry (Fig. 6). Cellular composition analysis showed that differentially phosphorylated proteins were mainly distributed in the postsynaptic density, followed by dendrites, neuronal cell bodies, and glutamatergic synapses in both the H vs. C comparison group and the S vs. H comparison group. Molecular function analysis showed that differentially phosphorylated proteins were involved in the same functions, including protein kinase binding, calmodulin binding, and actin binding, in both the H vs. C comparison group and the S vs. H comparison group. Biological process analysis showed that differentially phosphorylated proteins were mainly involved in axonogenesis both in the H versus C comparison group and in the S versus H comparison group.

**Fig. 7:**
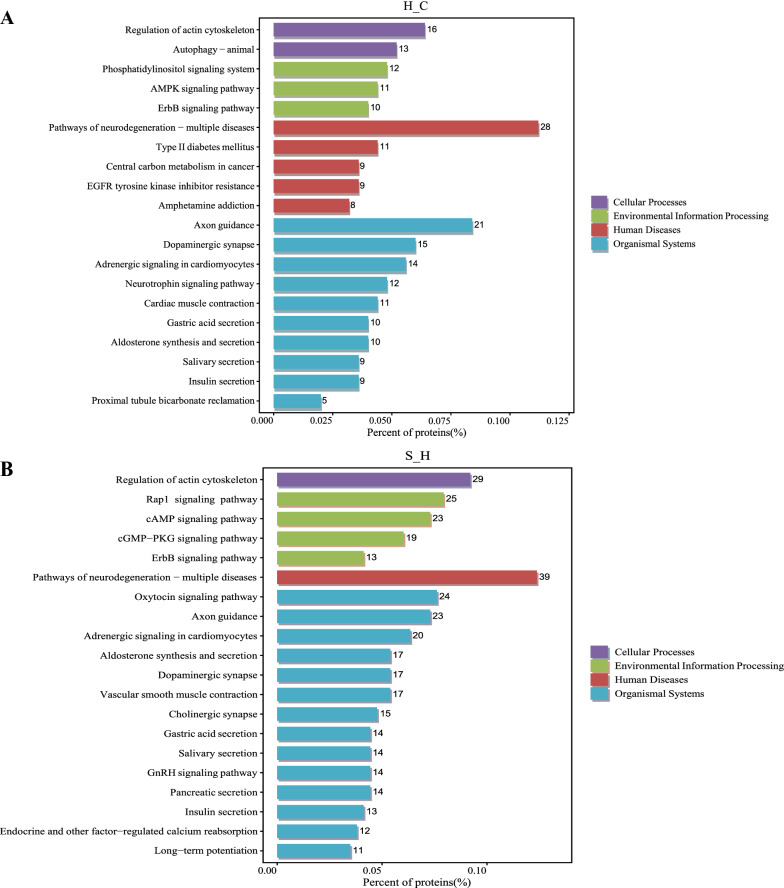
**A** KEGG enrichment analysis of proteins corresponding to the differential sites in **A** H/C and **B** S/H

**Fig. 8:**
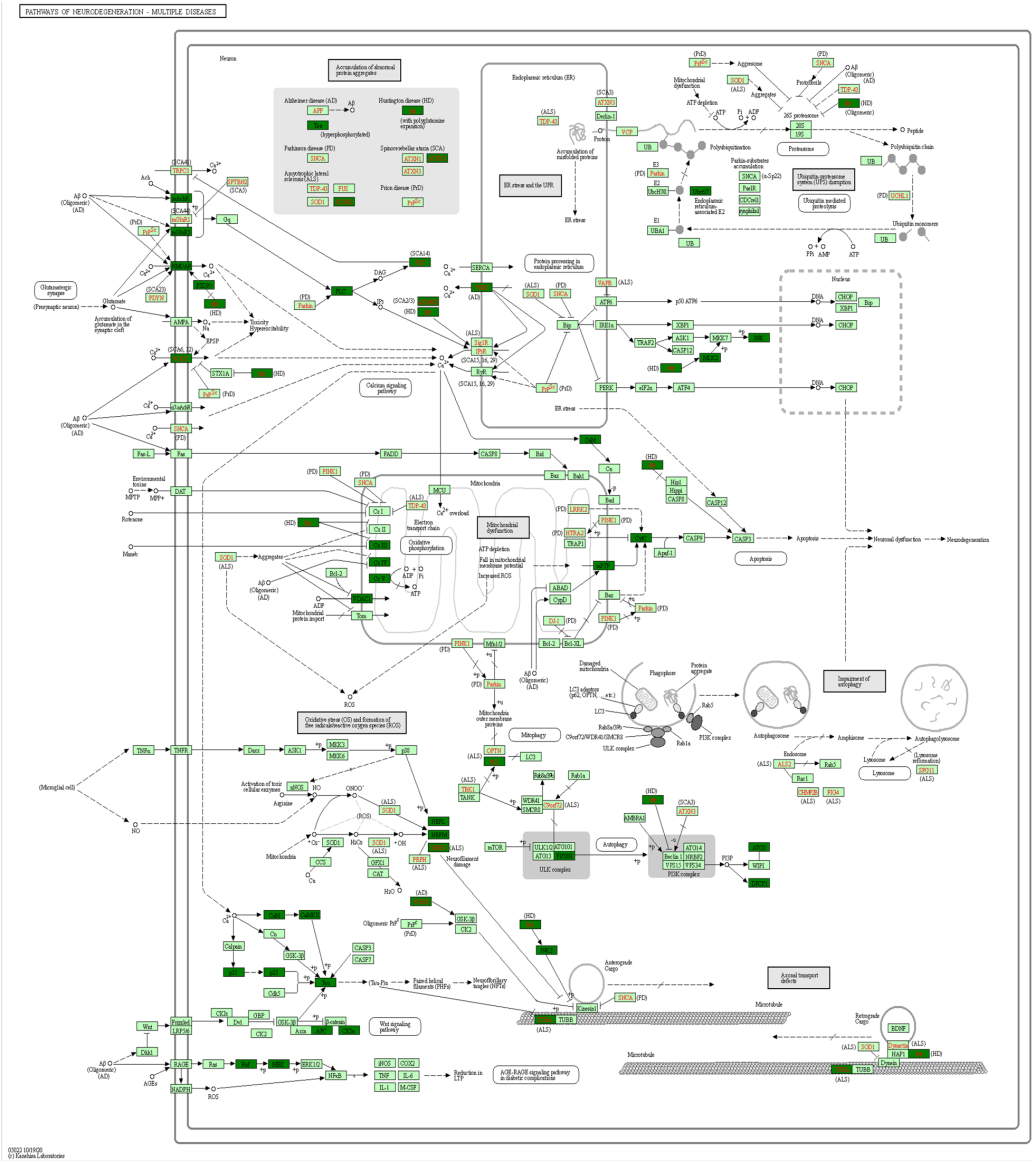
Pathways involved in -multiple neurodegeneration diseases

To explore potentially relevant pathways of semaglutide on high-fat diet-induced memory impairment, we performed KEGG pathway analysis on relevant targets. In total, 239, 249 and 255 biological pathways were annotated in differentially phosphorylated proteins in H:C, S:H and S:C, respectively. The top 20 significantly enriched pathways for the three groups are shown in Fig. 7. We also found that many pathways were enriched in the three comparative groups, including pathways of neurodegeneration-multiple diseases, axon guidance, regulation of actin cytoskeleton, dopaminergic synapse, adrenergic signaling in cardiomyocytes and ErbB signaling pathway. Notably, pathways of neurodegeneration-multiple diseases pathway showed the most involved targets. This pathway is shown in Fig. 8.

### Network pharmacology

**Fig. 9:**
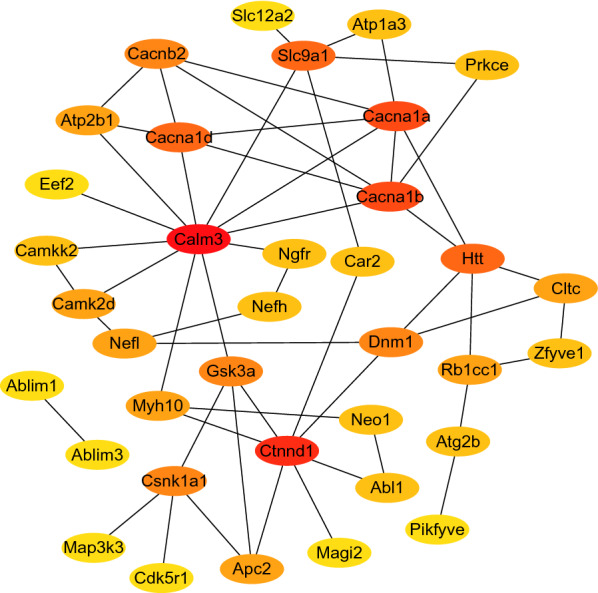
Protein–protein interaction networks. Protein–protein interaction networks with opposite direction of phosphorylation sites in the H/C and S/H groups and involved in the first 20 KEGG pathways

To further explore the mechanism by which semaglutide improves cognitive function, we performed network pharmacology. First, phosphorylated proteins in the H/C and S/H groups were screened using the principle of opposite direction (i.e., phosphorylated proteins were upregulated in the H/C group and downregulated in the S/H group or vice versa). In total, 44 target proteins were identified (Table 1). Next, the protein–protein Interaction (PPI) Network was constructed using the Cytoscape tool. Figure 9 shows the overall view of the relationships between the 36 targets (the other 8 genes are disconnected).

**Table 1:** Phosphorylated proteins reversed after semaglutide treatment

Gene name	Gene name	Description	H/C	S/H
P42859	Htt	Huntingtin	inf	0
P08551	Nefl	Neurofilament light polypeptide	0	inf
P19246	Nefh	Neurofilament heavy polypeptide	inf	0
Q6PHZ2	Camk2d	Calcium/calmodulin-dependent protein kinase type II subunit delta	inf	0
Q5SW75	Ssh2	Protein phosphatase Slingshot homolog 2	inf	0
Q8CC27	Cacnb2	Voltage-dependent L-type calcium channel subunit beta-2	inf	0.4062837662262003
P00920	Ca2	Carbonic anhydrase 2	inf	0
Q80U44	Zfyve1	Zinc finger FYVE domain-containing protein 16	inf	0
Q8C0T5	Sipa1l1	Signal-induced proliferation-associated 1-like protein 1	inf	0
Q9ESK9	Rb1cc1	RB1-inducible coiled-coil protein 1	inf	0
Q61084	Map3k3	Mitogen-activated protein kinase kinase kinase 3	inf	0
Q91VS8	Farp2	FERM, ARHGEF and pleckstrin domain-containing protein 2	inf	0
P39053	Dnm1	Dynamin-1	inf	0
P30999	Ctnnd1	Catenin delta-1	2.143379036683881	0
Q9CPQ1	Cox6c	Cytochrome c oxidase subunit 6C	inf	0
O70174	Chrna4	Neuronal acetylcholine receptor subunit alpha-4	inf	0
Q8C078	Camkk2	Calcium/calmodulin-dependent protein kinase kinase 2	inf	0
Q99246	CACNA1D	Voltage-dependent L-type calcium channel subunit alpha-1D	0	inf
P97445	CACNA1A	Voltage-dependent P/Q-type calcium channel subunit alpha-1A	0	inf
O55017	CACNA1B	Voltage-dependent N-type calcium channel subunit alpha-1B	0	inf
P0DP28	Calm3	Calmodulin-3	0	inf
G5E829	Atp2b1	Plasma membrane calcium-transporting ATPase 1	0	inf
Q61165	Slc9a1	Sodium/hydrogen exchanger 1	0	inf
Q6PIC6	Atp1a3	Sodium/potassium-transporting ATPase subunit alpha-3	0	2.108920584351263
Q8BTW9	Pak4	Serine/threonine-protein kinase PAK 4	0	inf
P55012	Slc12a2	Solute carrier family 12 member 2	0	inf
Q61879	Myh10	Myosin-10	0	inf
P00520	Abl1	Tyrosine-protein kinase ABL1	0	inf
P16054	Prkce	Protein kinase C epsilon type	0	inf
Q9JJZ4	Ube2j1	Ubiquitin-conjugating enzyme E2 J1	0	inf
Q9Z1T6	Pikfyve	1-phosphatidylinositol 3-phosphate 5-kinase	0	inf
Q9QYX7	Pclo	Protein piccolo	0	inf
Q9Z0W1	Ngfr	Tumor necrosis factor receptor superfamily member 16	0	inf
P97798	Neo1	Neogenin	0	inf
Q9WVQ1	Magi2	Membrane-associated guanylate kinase, WW and PDZ domain-containing protein 2	0	inf
P58252	Eef2	Elongation factor 2	0	inf
Q8BK63	Csnk1a1	Casein kinase I isoform alpha	0	inf
Q68FD5	Cltc	Clathrin heavy chain 1	0	inf
P61809	Cdk5r1	Cyclin-dependent kinase 5 activator 1	0	inf
Q80XK6	Atg2b	Autophagy-related protein 2 homolog B	0	inf
Q69ZX8	Ablim3	Actin-binding LIM protein 3	inf/0	0/inf
Q8K4G5	Ablim1	Actin-binding LIM protein 1	inf/0	0/inf
Q9Z1K7	Apc2	Adenomatous polyposis coli protein 2	inf/0	0/inf
Q2NL51	Gsk3a	Glycogen synthase kinase-3 alpha	inf/0	0/inf

## Discussion

Numerous clinical experiments and animal studies have shown that GLP-1 receptor agonists can exert neuroprotective effects on the hippocampus by modulating insulin signaling, improving mitochondrial function in the brain, and reducing inflammatory responses[3]. In addition, it can activate AMPK and PI3K/Akt signaling pathways and promote autophagy, thus improving cognitive function [36]. Studies have reported that the GLP-1 receptor agonist semaglutide is involved in appetite regulation, food intake, and gastric emptying [22]. Its therapeutic efficacy has been demonstrated in patients with type 2 diabetes, obesity and cardiovascular disease [17]. However, the effects and mechanisms of action of semaglutide on the hippocampal tissue and cognitive function in high-fat diet-induced obese mice have not been investigated. In this study, we analyzed hippocampal tissues of normal diet-fed mice or high-fat diet-fed mice treated with or without semaglutide using phosphorylated proteomics techniques and assays, and identified 7108 differentially phosphorylated sites corresponding to 2010 phosphorylated proteins.

In the results of the study, obesity model mice showed abnormalities in lipid metabolism, manifested by high TC, TG, LDL-C and HDL-C. After semaglutide intraperitoneal injection treatment, serum TG and LDL-C levels were significantly reduced (P < 0.05), HDL-C levels were decreased (P < 0.01) in mice. Recent studies have shown that both high and low HDL cholesterol are not good. Liu et al. [18] found that high levels of HDL-C were associated with high cardiovascular mortality in patients with high serum amyloid A (SAA). It has also been found that HDL-C metabolism, structure, function and levels can be profoundly altered under conditions of significant oxidative stress and chronic inflammation. These abnormalities not only result in impaired cholesterol transport, increased systemic oxidative stress, and endothelial dysfunction, but also lead to reduced HDL function and to the production of pro-inflammatory HDL-C, which increases the burden of atherosclerosis and cardiovascular disease [10]. In addition, our study found that the levels of IL-1, IL-6 and MDA were significantly higher (P < 0.05) and the levels of SOD were significantly lower (P < 0.01) in obese mice compared to the normal diet group. And the above indicators were reversed after semaglutide treatment. Therefore, we speculate that high-fat diet may lead to HDL dysfunction and promote the development of inflammation and oxidative stress. And semaglutide may improve inflammation, oxidative stress by reducing its level. However, the exact mechanism still needs further study.

This study also found that group H mice showed obesity and cognitive decline, and that semaglutide reduced weight and improved cognitive decline. Analysis of clinical data has shown that glucose-lowering agents (GLP1 receptor agonists, SGLT2 inhibitors, metformin and DPP4 inhibitors) reduce the risk of dementia in patients with diabetes and that this neuroprotective effect is independent of weight loss [35]. Therefore, we hypothesize that the benefits of semaglutide can be extended to patients with these cognitive impairments, but more clinical studies are needed in the future to further confirm this. Notably, the pathway enrichment results showed that semaglutide could regulate several pathways, including Pathways of neurodegeneration—multiple diseases, Axon guidance and Dopaminergic synapse. We identified 3 of the 44 overlapping proteins for further PPI analysis, including Huntington (HTT), neurofilament protein light chain (NEFL) and neurofilament protein heavy chain (NEFH) (Table 2). Notably, most of the differentially expressed phosphorylated proteins identified in this study were mainly involved in pathways of neurodegeneration—multiple diseases, such as Alzheimer's disease (AD), Parkinson's disease (PD), Huntington's disease and amyotrophic lateral sclerosis, suggesting that high-fat diet-induced obesity may share a common molecular mechanism with the above diseases.

**Table 2:** HTT, NEFL and NEFH are involved in pathways of neurodegeneration-multiple diseases

Group name	Gene name	Regulated type	P value	Positions within proteins	Amino acid	KEGG pathway
H/C	HTT	inf	3.89381E−06	1843	S	Pathways of neurodegeneration-multiple diseases
S/H	HTT	0	3.89381E−06	1843	S	Pathways of neurodegeneration-multiple diseases
H/C	NEFL	0	3.11066E−06	473	S	Pathways of neurodegeneration-multiple diseases
S/H	NEFL	inf	2.55318E−09	473	S	Pathways of neurodegeneration-multiple diseases
H/C	NEFH	inf	0.00203392	661	S	Pathways of neurodegeneration-multiple diseases
S/H	NEFH	0	1.59974E−06	661	S	Pathways of neurodegeneration-multiple diseases

HTT is a macromolecular protein (348 kDa) encoded by the Huntington gene that interacts with various proteins and plays an essential function during embryonic development, growth and development, as well as hematopoiesis and neurogenesis in living organisms [26]. Studies have shown that deletion in HTT gene prevents embryos from developing normally, leading to premature embryonic death and *Htt* gene knock out in adulthood induces neurodegenerative diseases [19, 25]. Wild-type *Htt* exerts anti-apoptotic effects and up-regulates gene transcription and transport of neurotrophic factors, thus maintaining normal neuronal functioning [16]. In addition, several recent studies have shown that normal HTT is associated with AD pathology. One study showed a higher frequency of intermediate CAG repeat alleles of HTT in AD patients [23]. Axenhus et al. [1] found elevated levels of Huntington protein in hippocampal autopsy samples from patients with Alzheimer's disease. They also established that HTT accumulation in AD brains may not be the result of mutant *Htt* or Poly Q amplification, but due to altered cellular activity, leading to subsequent increased HTT production, transport alterations. This suggests that HTT is a hallmark of AD pathology. Schedin-Weiss et al. [29] also confirmed through proteomic studies that HTT levels were elevated in the hippocampi of young App^NL −F/NL−F^ mice of different ages before the appearance of amyloid plaques, neuroinflammation and memory impairment. In our study, serine phosphorylation at site 1843 of HTT was increased in the hippocampi of group H mice, whereas HTT phosphorylation at Ser^1843^ was reduced after semaglutide intervention. Therefore, we speculated that obesity may impair hippocampal cognitive function by upregulating hippocampal HTT expression, whereas stigmasteride protects the hippocampus by downregulating HTT expression. However, the exact mechanism needs to be further investigated.

NEFL is a member of type IV intermediate filament family, which occurs in neurofilaments with the 200 kD NEFH and 160 kD neurofilament protein medium chain in a certain ratio [39]. However, unlike others, NEFL has a self-assembling subunit and is abundantly expressed in the white matter of axons and plays an important role in maintaining neuronal morphology and regenerating myelinated axons [2]. Previous reports have shown that abnormal expression of NEFL is associated with various neurodegenerative diseases such as Alzheimer's disease, Parkinson's disease, and amyotrophic lateral sclerosis [8, 9, 28]. It has been confirmed that mutations in the *Nefl* gene lead to abnormal aggregation of neurofilaments in the cytosol, disrupting the formation of normal reticular structures, causing impaired axonal transport and degenerative axonal degeneration, and ultimately leading to the development of peroneal muscular dystrophy [8]. In contrast, a study in APP/PS1 NEFL^−^/^−^ mice showed that deletion of the *Nefl* gene increased neocortical amyloid-β (Ab) deposition, synaptic vulnerability and microglia proliferation around Ab plaques, suggesting a protective role for NEFL in AD [9]. In addition, NEFL can act on postsynaptic density by anchoring NMDA receptors and NEFL mRNA and protein expression levels are reduced in postmortem brain regions in several schizophrenic patients [4]. Our study showed reduced serine phosphorylation at site 473 of NEFL in the hippocampus of group H mice. Reduced expression of NEFL has been associated with defective synaptic function and consequential behavioral impairment [8]. Interestingly, NEFL protein synthesis, which is essential for axonal integrity and synaptic function, was restored after semaglutide treatment, which attenuated cognitive dysfunction.

NEFH is the major cytoskeletal structural protein of neurons. Phosphorylated NEFH plays an important role in the protein’s subsequent binding to cytoskeletal proteins and other proteins and normal axonal nutrient transport [11]. The phosphorylation of NEFH in the axon may be regulated by a more sophisticated mechanism. Excessively phosphorylated or unphosphorylated NEFH may have adverse effects on neurons. Studies have shown that inhibition of NEFH phosphorylation impairs the formation of cross-linked bridges and disturbed neurofilament arrangement, which blocks axonal transport of various important components in neurons and ultimately leads to neuronal degeneration and death [38]. Effective binding of neurofilament proteins and cytoskeletal proteins in the axon is necessary for transportation of neuronal axonal material. while overphosphorylated NEFH would dissociate from the transporter and fail to bind to important components in the axoplasm, accumulating abnormally, thus reducing the efficiency of axoplasmic component. As a result, the transport efficiency of axoplasmic components is adversely affected, leading to neuronal damage [34]. In addition, when NEFH is abnormally phosphorylated in the cytoplasm, it may accumulate in the cytosol and fail to enter the axon to aid in material transport, leading to neuronal degeneration and death [21]. In the present study, increased serine phosphorylation at site 661 of NEFH in the hippocampus of group H mice suggested that high phosphorylation of NEFH may make decrease the axon diameter, inhibit axon growth, and reduce the speed of neurotransmission to affect cognitive function. This effect was reversed following semaglutide treatment.

However, there are limitations to our study. First, we did not screen for serum GLP-1 levels because our serum samples were used up during the experiment. Second, due to time and funding considerations, the screened proteins, their functions, and the involved pathways were not explored. We plan to elucidate this aspect in our next research to expand our understanding of the mechanism of action of semaglutide in obesity-related cognitive impairment.

In conclusion, this study demonstrates for the first time that semaglutide can maintain normal neurocytoskeletal structure, promote axonal growth and neurogenesis by downregulating the phosphorylation of HTT Ser^1843^ and NEFH Ser ^661^, and upregulating the phosphorylation of NEFL Ser^473^ thereby reducing the risk of developing obesity-induced cognitive impairment. These findings provide new insight into the pathogenesis of obesity-induced cognitive impairment. The proteins associated with these neuroprotective effects (HTT, NEFH and NEFL) are potential drug targets for the treatment of obesity-induced cognitive impairment with semaglutide.

## Data Availability

The data for this article can be found in the supplementary materials.
